# Activation of Dendritic Cells by the Novel Toll-Like Receptor 3 Agonist RGC100

**DOI:** 10.1155/2013/283649

**Published:** 2013-12-02

**Authors:** Kai Naumann, Rebekka Wehner, Anett Schwarze, Christiane Petzold, Marc Schmitz, Jacques Rohayem

**Affiliations:** ^1^Riboxx GmbH, Meissner Straße 191, 01445 Radebeul, Germany; ^2^Institute of Immunology, Medical Faculty Carl Gustav Carus, Dresden University of Technology, Fetscherstraße 74, 01307 Dresden, Germany; ^3^Institute of Virology, Medical Faculty Carl Gustav Carus, Dresden University of Technology, Fetscherstraße 74, 01307 Dresden, Germany

## Abstract

Toll-like receptor (TLR) 3 agonists emerged as attractive candidates for vaccination strategies against tumors and pathogens. An important mechanism of action of such agonists is based on the activation of TLR3-expressing dendritic cells (DCs), which display a unique capacity to induce and stimulate T-cell responses. In this context, it has been demonstrated that targeting of TLR3 by double-stranded RNA such as poly(I:C) results in potent activation of DCs. Major disadvantages of poly(I:C) comprise its undefined chemical structure and very poor homogeneity, with subsequent unpredictable pharmacokinetics and high toxicity. In the present study, we evaluated the physicochemical properties and biological activity of the novel TLR3 agonist RGC100. RGC100 has a defined chemical structure, with a defined length (100 bp) and molecular weight (64.9 KDa) and a good solubility. RGC100 is stable in serum and activates myeloid DCs through TLR3 targeting, as evidenced by gene silencing experiments. Activation of mouse and human myeloid CD1c^+^ DCs by RGC100 leads to secretion of several proinflammatory cytokines. In addition, RGC100 improves the ability of CD1c^+^ DCs to stimulate T-cell proliferation. Due to its physicochemical properties and its immunostimulatory properties, RGC100 may represent a promising adjuvant for prophylactic and therapeutic vaccination strategies.

## 1. Introduction

In the initial phase of infection, the innate immune system generates a rapid and potent inflammatory response. This response aims at blocking dissemination of the infectious agent, with subsequent activation of T cells and B cells that mount the acquired immune response against the pathogen [1]. Recognition of pathogen-related components by immune cells occurs through pathogen recognition receptors (PRR). PRRs are present on cell surfaces, in endosomes, or in cytosol. Toll-like receptors (TLR) represent an important family of PRRs [2, 3]. They are expressed on various subsets of immune cells such as dendritic cells (DCs) [4]. DCs are professional antigen-presenting cells that play an important role in the induction and maintenance of innate and adaptive immune responses [5, 6]. Due to their functional properties and prominent expression of Toll-like receptors, DCs represent promising candidates for TLR agonist-based vaccination strategies against tumors and pathogens [7, 8].

Expression of TLR3 has been evidenced in BDCA1^+^ myeloid DCs (mDCs), human-monocyte-derived DCs (MoDCs) but not in plasmacytoid DCs [9–13]. Double-stranded RNA (dsRNA) is a ligand of TLR3 [14]. It is recognized as a pathogen-associated molecular pattern (PAMP), triggering innate immune response through the interaction with TLR3 expressed by DCs [15–17]. Of note, a variety of cancer cells have been reported to express TLR3. Upon triggering of TLR3 in tumor cells, apoptosis and/or antitumoral effect occur [18–21].

Polyinosinic-polycytidylic acid poly(I:C) is a potent activator of innate immunity [14, 22]. Poly(I:C) activates DCs through combined targeting of various innate immunity pathways, including TLR3. Major disadvantages of poly(I:C) comprise its undefined chemical structure and very poor homogeneity, resulting from its manufacturing process [23]. Poly(I:C) is composed of a mix of single-stranded and double-stranded RNA molecules ranging from about 1.5 to 8 kb [22], imperfectly annealed as dsRNA or single-stranded RNA. This is mainly due to limited solubility and difficult reconstitution of poly(I:C) that requires heating (50–60°C) and slow cooling over many hours to achieve reannealing of both poly(I) and poly(C) strands. As a consequence, poly(I:C) has a reported toxicity in clinical trials, ranging from hypersensitivity to coagulopathy, renal failure, or systemic cardio-vascular failure [24]. A further problem of dsRNA compounds such as poly(I:C) are their rapid degradation in body fluids by RNAses, with a reported half-life of few minutes [25, 26] and subsequent unpredictable pharmacokinetics of degradation products. Optimization of physicochemical properties of poly(I:C) has led to generation of derivatives that have increased stability in body fluids (such as polyICLC), [27] or reduced toxicity through reduced stability in body fluids (such as poly(I:C_12_U) [28, 29]. Poly(I:C) and its derivatives are produced under GMP conditions for intravenous administration and have been tested in various clinical trials [28–30].

In the present study, structure, analytical profile and biological activity of the novel TLR3 agonist RGC100 are presented. RGC100 displays a very well defined chemical structure, length and molecular weight, a good solubility and serum stability, being able to activate DCs in a dose-dependent manner by specifically targeting endosomal TLR3.

## 2. Materials and Methods

### 2.1. Physicochemical Analysis

Analysis of RGC100 length and integrity was performed on 12% native PAGE. DNA marker (Fermentas, Germany) was used to illustrate molecular size distribution and RNA staining was achieved by using Stains-all (Alfa Aesar, USA). Analysis of poly(I:C) was performed on 1% native agarose gel electrophoresis. Two different poly(I:C) compounds were used: poly(I:C) with a low molecular weight (LMW, 0.2–1 kb, Invivogen, USA), and poly(I:C) with a high molecular weight (HMW, 1.5–8 kb, Invivogen, USA). RNA marker (Promega, Germany) was used to illustrate molecular size distribution.

Physical characterization of RGC100 in solution was performed by size-exclusion chromatography (SEC) with UV, refractive index (RI), and right angle light scattering (RALS) detection on the Viscotek TDAmax (Malvern, UK). A sample volume of 125 *μ*L (~100 *μ*g RGC100) was injected to a Superdex 200 10/300 column (GE Healthcare, USA) and SEC was performed by using phosphate buffer saline.

### 2.2. Immunomagnetic Isolation of CD1c^+^ DCs and CD3^+^ T Lymphocytes

Blood samples were obtained with informed consent from healthy donors. The study was approved by the institutional review board of the University Hospital of Dresden (no. EK 27022006). Peripheral blood mononuclear cells (PBMCs) were obtained by Ficoll-Hypaque (Biochrom, Germany) density centrifugation. Subsequently, human CD1c^+^ DC were isolated from freshly prepared PBMCs by immunomagnetic negative depletion and positive selection according to the manufactures instructions (Miltenyi Biotec, Germany). CD3^+^ T cells were isolated from PBMCs by negative depletion using immunomagnetic separation according to the manufacturer's instructions (Miltenyi Biotec, Germany). To analyze the purity of the cell preparations, CD1c^+^ DCs were stained with PE-conjugated anti-CD1c^+^ and FITC-conjugated anti-CD19 antibodies and CD3^+^ T cells with PE-conjugated anti-CD2 and FITC-conjugated anti-CD3 antibodies. Purity was determined by FACS analysis, which was performed on a FACSCalibur flow cytometer (BD Biosciences, Heidelberg, Germany).

### 2.3. T-Cell Proliferation Assay

CD1c^+^ DCs (1 × 10^4^/well) were cocultured with autologous CD3^+^ T cells (1 × 10^5^ cells/well) in the presence or absence of 50 *μ*g/mL RGC100 or poly(I:C) in round-bottomed 96-well plates. Before coculture, T-cells were stained with 1 *μ*M cell proliferation dye eFluor 670 (eBioscience, Germany). Cells were incubated for 8 days, harvested and T cell proliferation was analyzed by flow cytometry, which was performed on a FACSCalibur flow cytometer (BD Biosciences).

### 2.4. Cells and Cytokine Assays

JAWS II cell line was obtained from American Type Culture Association (ATCC, USA). JAWS II is an immortalized immature myeloid DC line derived from C57BL/6 mice, which displays a similar phenotypic profile as resting bone-marrow-derived DCs (BMDCs) [31]. Cells were plated in round-bottomed 96-well plates at 5 × 10^4^/well in DMEM with 10% fetal calf serum (FCS) and 1% penicillin/streptomycin (100 U/mL). Cells were incubated with RGC100 (Riboxx, Germany) at different concentrations with or without preincubation with chloroquine (Invivogen, USA). After 16 h, supernatants were collected and the concentration of cytokines and chemokines was determined by ELISA according to manufacturer's instructions (Qiagen, Germany).

To assess the toxicity of siRNA on JAWs II DCs, a cell proliferation assay was performed as previously described [32]. Briefly, JAWS II DCs were seeded into a 96-well plate with 5 × 10^4^ cells/well. A serial dilution of siRNA at a concentration ranging from 200 nM to 12.5 nM was added to the cells. After 24 h, 100 *μ*L of MTS solution (3-(4,5-dimethylthiazol-2-yl)-5-(3-carboxymethoxyphenyl)-2-(4-sulfophenyl)-2H-tetrazolium) was added to each well. After 2 h, absorbance of the solution was measured at 490 nm and the CC_50_ value was determined.

Human CD1c^+^ DCs were plated in round-bottomed 96-well plates at 2.5 × 10^4^/well in RPMI 1640 medium containing 10% human AB serum (CCPRO, Germany), 2 mM L-glutamine, 1% nonessential amino acids, 100 U/mL penicillin, and 100 mg/mL streptomycin (Biochrom, Germany). Then, cells were stimulated with RGC100 or poly(I:C) (Sigma-Aldrich, Deutschland) at different concentrations. After 24 h, supernatants were collected and the concentration of IL-1*β*, IL-6, and TNF-*α* was determined by ELISA according to the manufacturer's instructions (BD Biosciences).

### 2.5. Gene Silencing of TLR3

Gene silencing was performed using IBONI siRNA (Riboxx, Germany) targeting TLR3 (5′-CTCGGCCTTAATGAAATTGAA-3′) and a nontargeting siRNA (Riboxx, Germany). Therefore, JAWS II cells were plated in round-bottomed 96-well plates at 5 × 10^4^/well and incubated at 37°C (5% CO_2_) for 16 h. Then, IBONI siRNA (Riboxx. Germany) was mixed to riboxxFECT transfection reagent (Riboxx, Germany) according to manufacturer's instructions and the mix was added to the wells at a concentration of 20 nM. At 6 h after transfection, RGC100 was added and the cells were incubated for 16 h. Subsequently, cells and supernatants were harvested. RNA was extracted from cells using the RNeasy kit (Qiagen, Germany) and used for subsequent qRT-PCR. Supernatants were used for cytokines measurement with ELISA according to the manufacturer's instructions (Qiagen, Germany). qRT-PCR was performed on LightCycler using QuantiTect Primer assays (Qiagen, Germany) for mouse TLR3 and mouse *β*-actin and QuantiTect SYBR Green RT-PCR kits (Qiagen, Germany) according to instructions of manufacturer.

### 2.6. Serum Stability Assays

RGC100 (1.6 *μ*M) was incubated in 80% FCS, mouse serum or human serum at 37°C from 1 h to 7 days. FCS and mouse serum were purchased Invitrogen (Germany) and Sigma-Aldrich (Germany) respectively. Human serum was collected from a blood donor. Integrity of RGC100 was assessed through analysis on native 12% PAGE. As an indicator, RGC100 not incubated in serum was used. As a control for effective degradation of dsRNA by serum nucleases, a dsRNA (1.6 *μ*M) of 25 bp was incubated in FCS, mouse serum or human serum for 5 h and analyzed on native 12% PAGE.

### 2.7. Melting Point Analysis

Analysis of RGC100 (3 *μ*M) was performed on a LightCycler (Roche, Switzerland) using the riboxx LIGHT Kit (Riboxx, Germany) according to manufacturer's instructions.

### 2.8. Statistical Analysis

Student's *t*-test was performed to evaluate the significance of the results. Values of *P* < 0.05, were considered as significant.

## 3. Results and Discussion

### 3.1. RGC100 Has Defined Chemical Structure and Good Solubility

Design of RGC100 was performed based on the knowledge of structural and biological characteristics of TLR3 agonists. Crystal structure of the ectodomain of TLR3 with its dsRNA ligand [33] have shown that dsRNA of ~45 bp in length is sufficient for activation of TLR3 [34]. Most importantly, interaction of TLR3 ectodomains occurs only with the ribose backbone, indicating that triggering of TLR3 is not RNA sequence specific [33]. Hence, length of dsRNA is the major determinant of TLR3 triggering [35].

The choice of sequence composition of RGC100 was based on previous studies on biological activity *in vivo* of a polyguanidinic-polycytidinic compound (poly(G:C)). Poly(G:C) has been reported to have the same interferon-inducing and antiviral activity as poly(I:C) [36]. Importantly, poly(G:C) displays an up to 12.7-fold higher LD_50_ in comparison to poly(I:C) when administrated intravenously to mice (200 mg/kg versus 15.8 mg/kg, resp.) [36]. In rabbits, the LD_50_ of poly(G:C) administrated intravenously is up to 4.5 fold higher than poly(I:C) (1 mg/kg versus 0.22 mg/kg, resp.) [36].

**Figure 1:**
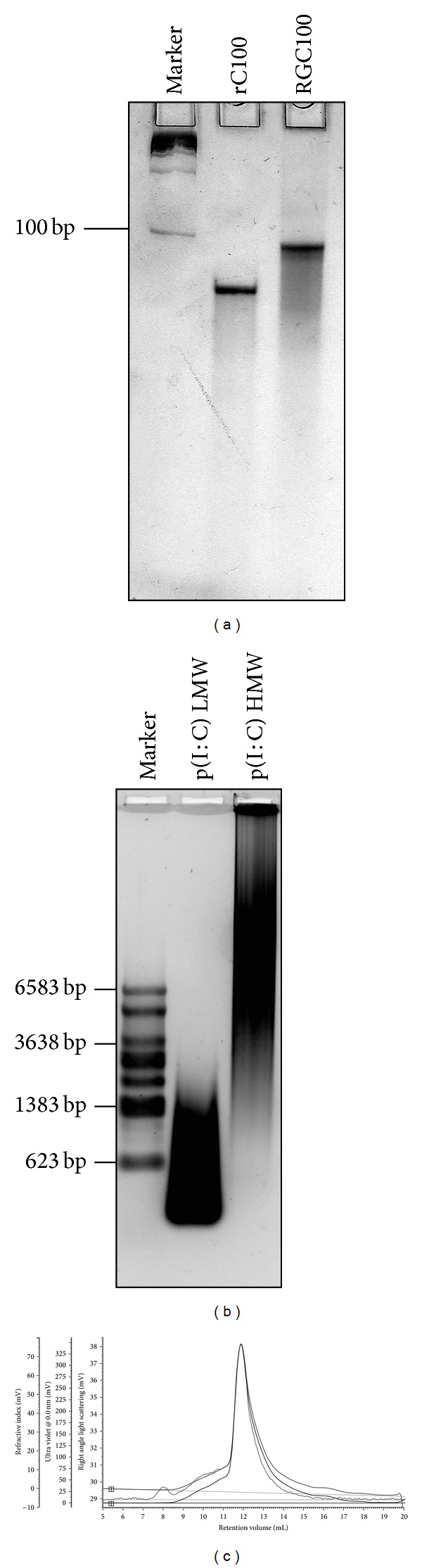
Determination of physicochemical properties of RGC100. (a) Analysis of RGC100 by 12% native PAGE. RGC100 displays a length of 100 bp as indicated. It consists of 100 rC bases paired to 100 rG bases, perfectly annealed in a double strand. As a reference, a 100 mer consisting of homopolymeric cytidine is shown. (b) Analysis of poly(I:C) by 1% native agarose gel electrophoresis. p(I:C) LMW: poly(I:C) with a low molecular weight. p(I:C) HMW: poly(I:C) with a high molecular weight. (c) Analysis of RGC100 by size-exclusion chromatography (SEC) with UV, RI and RALS detection. Data analysis provides information about molecular size and polydispersity (Mw/Mn = 1.015).

Taking these experimental observations on length and sequence composition into consideration, we have designed RGC100 that bears a length of 100 bp, and consists of 100 rC paired to 100 rG. Analysis by native PAGE and SEC with UV, RI and LS detection showed that RGC100 displays a defined physicochemical structure. RGC100 has an observed molecular weight of 64.6 kDa (MW_calc_ = 64.9 kDa) with low polydispersity (Figures 1(a) and 1(c)). Melting point of RGC100 was 91.6°C.

The defined chemical structure and good solubility of RGC100 are of importance to reduce potential toxic effects of TLR3 agonists. As observed for poly(I:C), the homogeneity of the compound plays an essential role in the genesis of toxicity. Poly(I:C) is a polydisperse and heterogeneous compound (Figure 1(b)) due to its polymeric macromolecular structure, being a mixture of single poly(rI) and poly(rC) as well as dsRNAs poly(I:C) of different lengths. This high chemical heterogeneity induces unpredictable pharmacokinetics [35] that translate into severe toxic side effects observed in clinical trials, such as coagulopathies, hypersensitivity reactions, renal failure, and even chock [24]. Heterogeneity of chemical structure of poly(I:C) leads to uncontrolled and combined signaling of at least three innate immunity pathways, namely TLR3, RIG-I, and/or MDA-5 [22]. Indeed, signaling of TLR3 is triggered by dsRNA with a length of more than 50 bp [34, 35], whereas signaling of RIG-I is stimulated by dsRNA of a length of 300–1000 bp [22, 37], and signaling of MDA-5 is activated by dsRNA of more than 1000 bp [22, 37, 38]. Moreover, the presence of ssRNA in the mixture resulting from imperfect annealing triggers TLR7 [33]. Taken together, the toxicity of poly(I:C) relates mainly to its heterogeneous composition and undefined chemical structure, with subsequent unpredictable pharmacokinetics and biological activity [35].

The advantages of using a TLR3 agonist such as RGC100 displaying defined physicochemical properties such as solubility and homogeneity, as well as precise chemical structure, length and molecular weight have been already highlighted by others [35]. An additional important advantage of RGC100 is the ability to fine-tune its potency for immune cell activation, by varying the length of the dsRNA compound. As reported previously [35], activation of TLR3 pathway *in vivo* depends mainly on the length of dsRNA, not the nucleotide sequence. Hence, RGC100 offers in addition to a highly defined chemical structure, the possibility to optimize the selectivity index as to immunological potency and toxicity for a specific indication. These physicochemical and functional properties differentiate RGC100 clearly form poly(I:C) (Table 1).

### 3.2. RGC100 Is Stable in Serum

**Figure 2:**
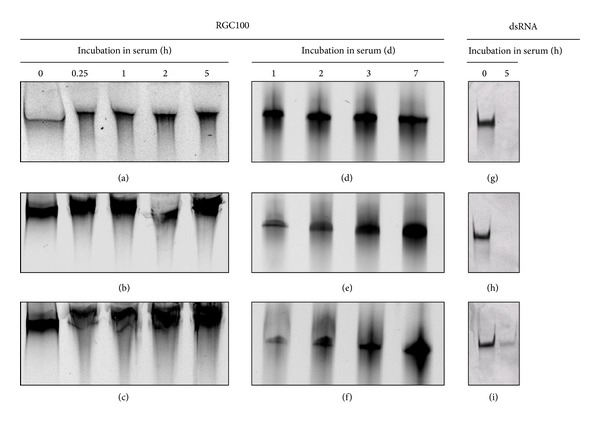
Assessment of the stability of RGC100 in FCS, mouse serum, or human serum. RGC100 (1.6 *μ*M) was incubated in 80% serum at 37°C and samples were analyzed on 12% native PAGE at indicated time points (h, hours and d, days). The corresponding untreated dsRNA (0 h) is shown as a reference. (a) Incubation of RGC100 in FCS up to 5 h. (b) Incubation of RGC100 in mouse serum up to 5 h. (c) Incubation of RGC100 in human serum up to 5 h. (d) Incubation of RGC100 in FCS up to 7 days. (e) Incubation of RGC100 in mouse serum up to 7 days. (f) Incubation of RGC100 in human serum up to 7 days. (g) Incubation of dsRNA (25 bp) used as a control in FCS for 5 hours. (e) Incubation of dsRNA (25 bp) used as a control in mouse serum for 5 hours. (f) Incubation of dsRNA (25 bp) used as a control in human serum for 5 hours.

Stability of RGC100 was examined in serum. RGC100 was incubated with FCS, mouse serum and human serum, and its integrity was assessed on native PAGE over time. As shown in Figures 2(a) to 2(f), RGC100 is stable in FCS, mouse and human serum up to 7 days. In contrast, a dsRNA of 25 bp used as a control is degraded in 5 h (Figures 2(g) to 2(i)), and poly(I:C) incubated with FCS was completely degraded in less than 5 minutes (see Supplemental Figure S1) available online on http://dx.doi.org/10.1155/2013/283649. This is not surprising, because as reported by others, dsRNA is usually degraded in serum within minutes [25, 26]. Degradation is due to nuclease activity of serum RNAses [25, 26]. The increased stability of RGC100 in serum can be explained by its GC content of 100%. Experimental data has shown that GC rich sequences display tight base stacking in dsRNA structure [39], with subsequent increase in duplex stability [39]. Increasing stability of duplex has been shown to increase half-life of dsRNA in serum. For instance, locked nucleic acids that increase stability of duplex confer resistance of dsRNA to serum nucleases [40]. Also, chemical modifications in ribose backbone (i.e., 2′-O-Methyl) that increase duplex stability improves its resistance to serum nucleases [25]. Our findings suggest that RGC100 displays physical properties making it suitable for *in vivo* applications.

### 3.3. RGC100 Activates Mouse Myeloid DCs

DCs display an extraordinary capacity to induce and expand CD8^+^ cytotoxic T lymphocytes (CTLs) and CD4^+^ T cells [8, 41]. CD8^+^ CTLs efficiently destroy tumor cells, whereas CD4^+^ T cells promote the antigen-presenting capacity of DCs and provide help for the activation and proliferation of CD8^+^ CTLs. Besides their unique ability to induce and stimulate T-cell responses, DCs efficiently improve the immunomodulatory and cytotoxic potential of natural killer cells, which essentially contribute to the elimination of virus-infected and tumor cells [42]. Due to their various immunostimulatory properties, DCs evolved as promising candidates for vaccination strategies against tumors and pathogens [7, 43].

**Figure 3:**
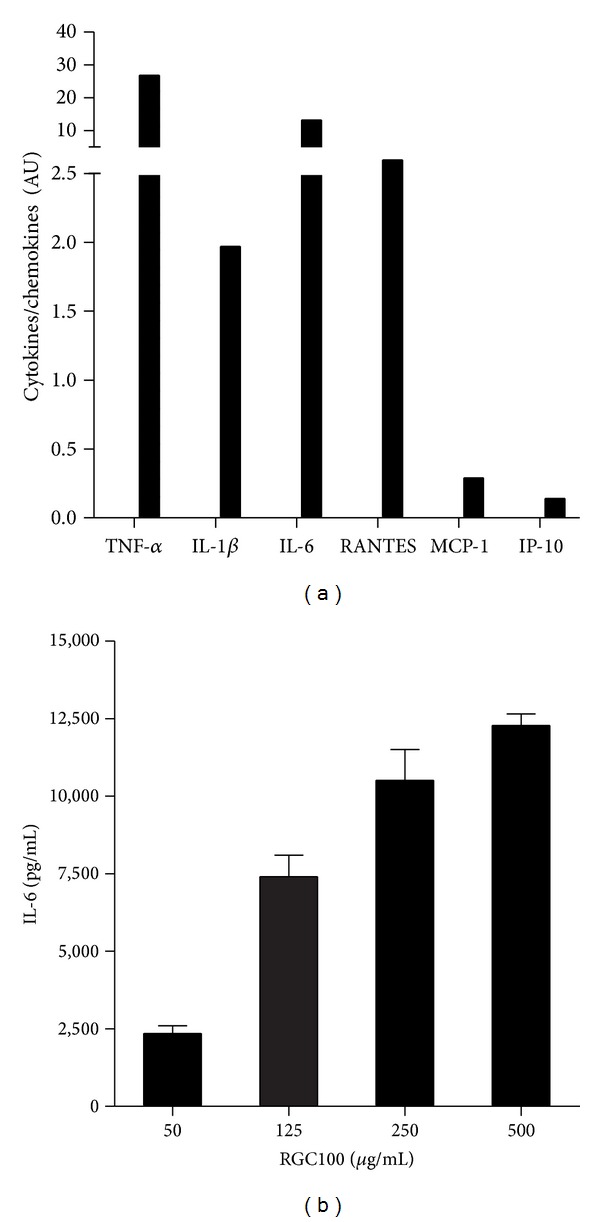
Activation of JAWS II DCs by RGC100 in a dose-dependent manner. (a), cytokine and chemokine profile of JAWS II DCs activated by RGC100. RGC100 was incubated with JAWS II DCs for 16 h at a concentration of 250 *μ*g/mL. Secretion of cytokines was measured by ELISA. Negative control consists of supernatant of cells incubated in the absence of RGC100. Values of negative control have been subtracted from the values represented on the graph. Values shown are mean ± SEM of two independent measures. (b), Dose-dependent activation of JAWS II DCs by RGC100. RGC100 was incubated with cells for 16 h at the indicated concentrations. Secretion of IL-6 was measured by ELISA. Values shown are mean ± SEM of two independent measures. Negative control consists of supernatant of cells incubated in the absence of RGC100. Values of negative control have been subtracted from the values represented on the graph.

In the present study, we investigated the impact of RGC100 on TLR3-expressing murine JAWS II cells, representing immature myeloid DCs, which have been used in studies focusing on antitumor and pathogen-specific immunity [31]. JAWS II cell line was expanded immediately after reception from supplier and frozen in aliquots. Cells were maintained in culture no more than 4 weeks. These procedures were done in order to ensure that no phenotypic drift occurs, as recommended by others [44]. As shown in Figure 3(a), incubation of JAWS II DCs with RGC100 resulted in secretion of various cytokines and chemokines. High levels of TNF-*α*, IL-6, and IL-1*β* were observed, as expected for activation of DCs. However, upon stimulation of JAWS II cells by RGC100, secretion of Type I interferon was not detected. In a further step, dose-response profiles of RGC100 were examined. As shown in Figure 3(b), efficient activation of JAWS II DCs was achieved over a range of concentrations from 50 to 500 *μ*g/mL.

### 3.4. RGC100 Is a Ligand of Endosomal TLR3

**Figure 4:**
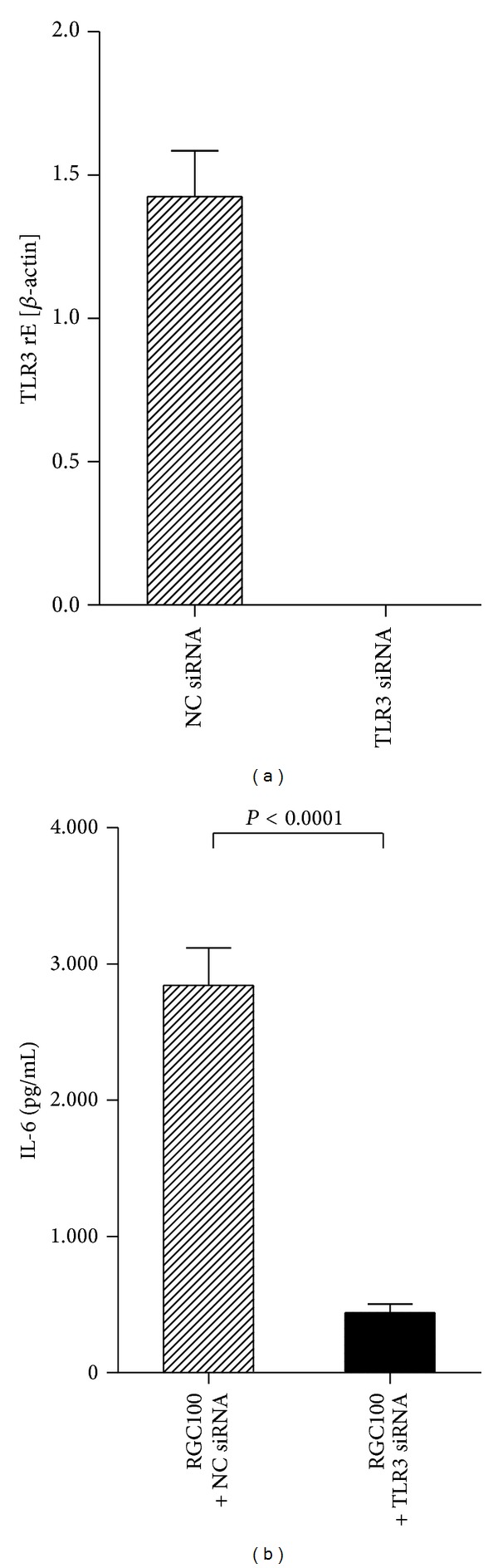
Inhibition of activation of JAWS II DCs by RGC100 using siRNA targeting TLR3. Cells were treated with siRNA targeting TLR3, then incubated with RGC100 at the indicated concentrations. RNA was extracted and supernatant was harvested. (a) Relative expression of TLR3 mRNA in cells treated with siRNA targeting TLR3 or with a nontargeting siRNA (NC siRNA). mRNA levels were normalized to *β*-actin mRNA. rE, relative expression. (b) Secretion of IL-6 was measured by ELISA. Values shown are mean ± SEM of two independent measures. Negative control consists of supernatant of cells incubated in the absence of RGC100 and siRNA. Values of negative control have been subtracted from the values represented on the graph.

To explore the mechanism of action of RGC100, knockdown of TLR3 in JAWS II DCs was performed. In order to prevent cytotoxicity resulting from off-target effects of siRNA, the CC_50_ of the siRNA was assessed in a cell proliferation assay. The CC_50_ of siRNA was >200 nM. Consequently, the concentration used to knockdown TLR3 (20 nM) was chosen to be 10-fold lower than the CC_50_. Additionally, the siRNA used in this assay displays a specific design that prevents off-target effects, as previously reported [45]. As shown in Figure 4, silencing of TLR3 expression in JAWS II DCs inhibited activation by RGC100, indicating that TLR3 is the ligand of RGC100.

**Figure 5:**
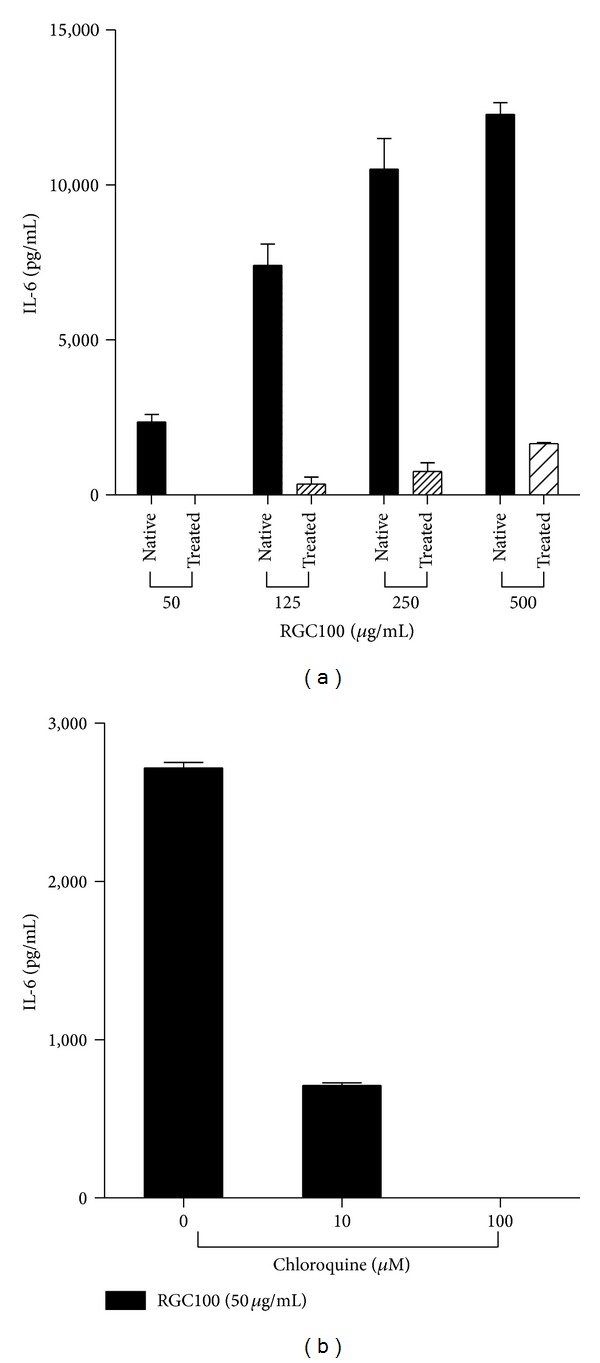
Inhibition of activation by RGC100 of JAWS II DCs through chloroquine. (a) Cells were first treated with chloroquine (treated, 100 *μ*M) or not (native), then incubated with RGC100 at the indicated concentrations. (b) Cells were first treated with chloroquine at the indicated concentrations, then incubated with RGC100 (50 *μ*g/mL). Secretion of IL-6 was measured in supernatant by ELISA. Values shown are mean ± SEM of two independent measures. Negative control consists of supernatant of cells incubated in the absence of RGC100. Values of negative control have been subtracted from the values represented on the graph.

In a further step, we examined whether endosomal acidification is essential for activation of JAWS II DCs by RGC100. For this purpose, cells were treated by chloroquine followed by incubation with RGC100. As shown in Figure 5(a), inhibition of the endosomal acidification by chloroquine impaired stimulation of JAWS II DCs by RGC100 in a dose-dependent manner (Figure 5(b)), indicating that endosomal uptake is essential for activation by RGC100.

### 3.5. RGC100 Activates Human Myeloid Dendritic Cells

**Figure 6:**
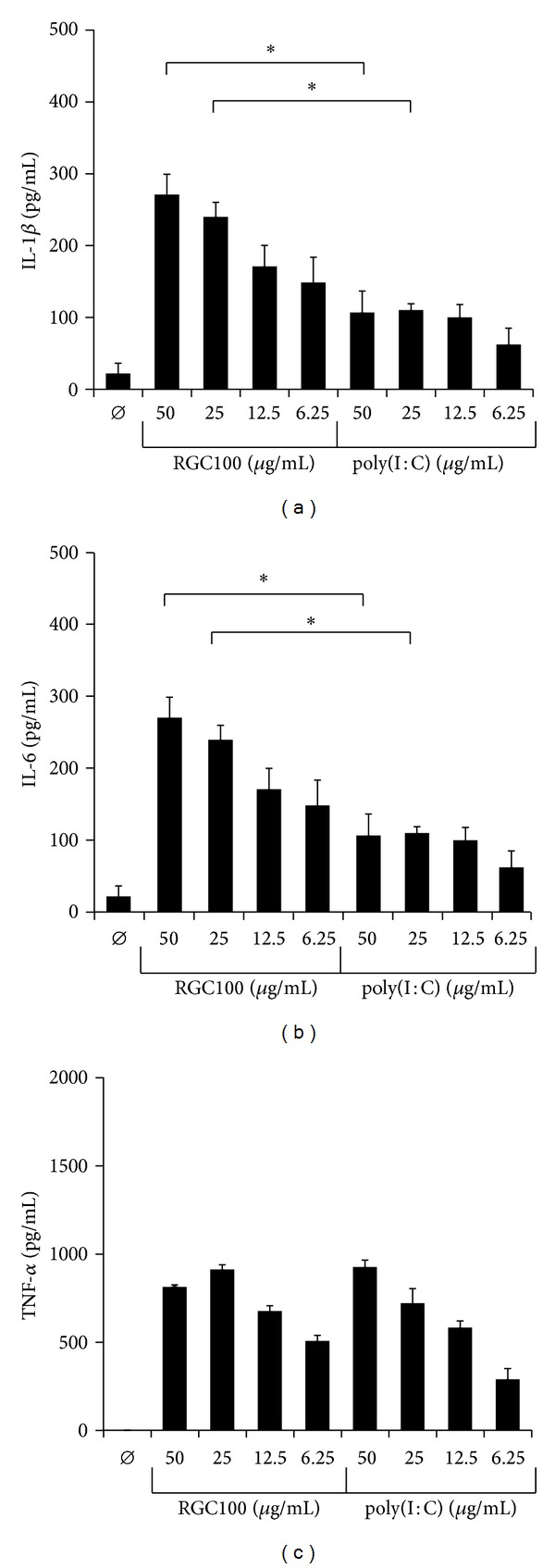
Activation of human myeloid CD1c^+^ DCs by RGC100 and poly(I:C). Freshly isolated CD1c^+^ DCs were cultivated in the presence or absence of RGC100 or poly(I:C). After 24 h, supernatants were harvested and the concentration of IL-1*β*, IL-6, and TNF-*α* was determined by ELISA as indicated. The results of one representative donor out of three performed with similar results are demonstrated. Values represent the mean ± SEM of triplicate samples and asterisks indicate a statistically significant difference (*P* < 0.05).

**Figure 7:**
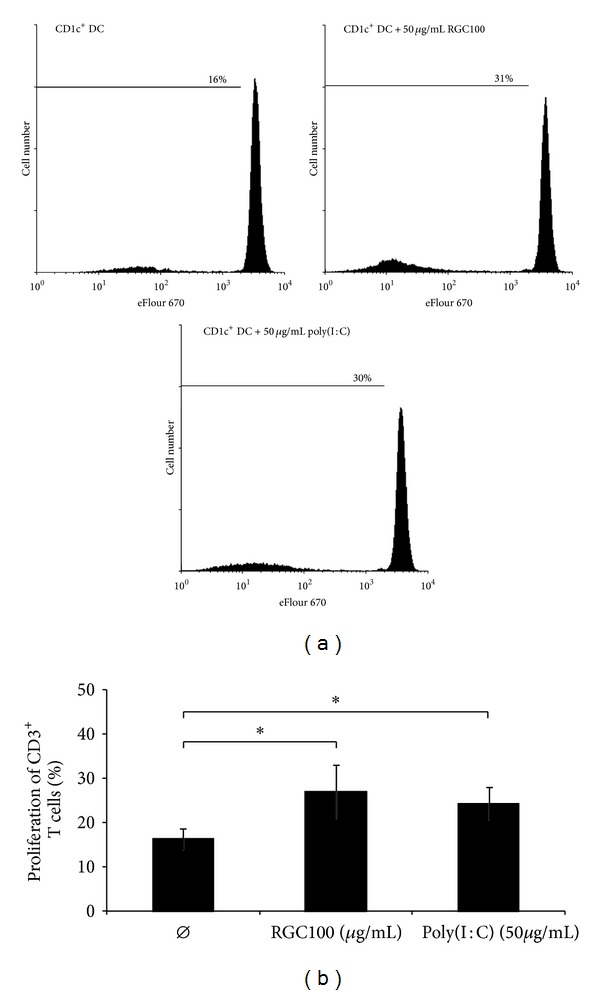
Impact of RGC100 and poly(I:C) on CD1c^+^ DC-mediated T-cell proliferation. CD1c^+^ DCs were cocultured with autologous T cells in the presence or absence of 50 *μ*g/mL RGC100 or poly(I:C). Before coculture, T cells were stained with cell proliferation dye eFluor 670. Cells were incubated for 8 days and harvested and T cell proliferation was determined by flow cytometry. (a) The results of one representative donor out of three performed with similar results are depicted. (b) The results of three different donors are presented as mean ± SEM. Asterisks indicate a statistically significant difference (*P < 0.05).

To get novel insights into the impact of RGC100 on the immunostimulatory properties of TLR3-expressing native human DCs, we investigated whether RGC100 promotes the release of proinflammatory cytokines by CD1c^+^ DCs in comparison to poly(I:C). Therefore, CD1c^+^ DCs and CD3^+^ T cells were immunomagnetically isolated from blood of healthy donors and maintained in the presence or absence of RGC100 and poly(I:C). The purity of isolated CD1c^+^ DC and CD3^+^ T cells was >90% as assessed by flow cytometric analysis (supplemental Figure  S2). As shown in Figure 6, both TLR-3 agonists efficiently stimulate the production of IL-1*β* and IL-6 by CD1c^+^ DCs. Interestingly, compared to poly(I:C), RGC100 has a significantly enhanced capacity to promote IL-1*β* and IL-6 by CD1c^+^ DCs, whereas the ability to stimulate TNF-*α* secretion was comparable between the TLR-3 agonists (Figure 6). In further experiments, we evaluated the impact of RGC100 on the ability of CD1c^+^ DCs to stimulate the proliferation of T cells. As depicted in Figure 7, RGC100 and poly(I:C) displayed a similar potential to augment CD1c^+^ DC-mediated T cell proliferation. These results indicate that the novel TLR3 agonist RGC100 efficiently stimulates the release of TNF-*α*, IL-1*β* and IL-6 by CD1c^+^ DCs and improves their capacity to promote T cell proliferation.

## 4. Conclusions

In the present study, experimental data on physicochemical properties and biological activity of the novel TLR3 agonist RGC100 are presented. RGC100 has optimal physicochemical properties, such as defined chemical structure and stability in serum. RGC100 activates murine myeloid DCs through targeting of endosomal TLR3, resulting in secretion of pro-inflammatory cytokines in a dose-dependent manner. In addition, RGC100 efficiently augments the secretion of pro-inflammatory cytokines by native human CD1c^+^ DCs and improves their capacity to promote T-cell proliferation. Based on these properties, RGC100 may represent a promising candidate for prophylactic and therapeutic vaccination strategies against tumors and pathogens.

## Supplementary Material

Supplemental Figuer 1. Assessment of the stability of poly(I:C) in FCS:
Poly(I:C) (2 *µ*g) was incubated in 80% serum at 37°C and samples were analyzed on 1% agarose gel and visualized by UV transillumination after staining with GelRed at indicated time points (min).
Supplemental Figuer 2. Purity of immunomagnetically isolated CD1c^+^ DCs and CD3^+^ T cells:
Human CD1c^+^ DC and CD3^+^ T cells were isolated from freshly prepared PBMCs of healthy donors by immunomagnetic separation. The purity of (A) CD1c^+^ DCs or (B) CD3^+^ T cells from one representative healthy donor out of (A) six or (B) performed with similar results are shown.

## Figures and Tables

**Table 1:** Comparison of the physicochemical and functional properties of RGC100 and poly(I:C).

	Length	Molecular weight	Chemical structure	Biostability^a^	Agonist of
RGC100	100 bp	64.6 KDa	dsRNA	7 days	TLR3
Poly(I:C)	~1500–8000 bp	~1020–5440 KDa	dsRNA ± ssRNA	<5 minutes	TLR3, RLRs^b^

^a^Biostability measured as resistance to serum nuclease; ^b^RLR: RIG-I-like receptors.
