# Supplementary figures and images for: Activation of Dendritic Cells by the Novel Toll-Like Receptor 3 Agonist RGC100
Source: Clin Dev Immunol. 2013 Dec 2;2013:283649. doi: 10.1155/2013/283649 (PMC3878805; doi:10.1155/2013/283649)

# Fig. S1

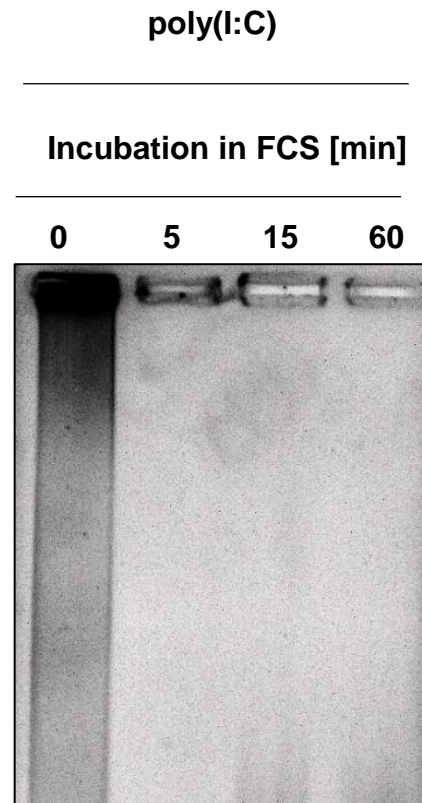

Supplement: Supplementary file 1 — Supplemental Figuer 1. Assessment of the stability of poly(I:C) in FCS: Poly(I:C) (2 µg) was incubated in 80% serum at 37°C and samples were analyzed on 1% agarose gel and visualized by UV transillumination after staining with GelRed at indicated time points (min). Supplemental Figuer 2. Purity of immunomagnetically isolated CD1c+ DCs and CD3+ T cells: Human CD1c+ DC and CD3+ T cells were isolated from freshly prepared PBMCs of healthy donors by immunomagnetic separation. The purity of (A) CD1c+ DCs or (B) CD3+ T cells from one representative healthy donor out of (A) six or (B) performed with similar results are shown. [file 283649.f1.pdf]
